# Investigation into the Influence of the Process Parameters on the Stability of a Poly(Vinyl)-Alcohol-Based Coating System

**DOI:** 10.3390/molecules29020386

**Published:** 2024-01-12

**Authors:** Viviana Claudia Canale, Lorenzo Paleari, Mario Bragaglia, Greta Petrella, Leonardo Severini, Francesca Nanni, Claudia Mazzuca, Antonio Palleschi

**Affiliations:** 1 Department of Chemical Science and Technologies, University of Rome ‘Tor Vergata’, Via della Ricerca Scientifica, 00133 Rome, Italy; viviana.claudia.canale@uniroma2.it (V.C.C.); petrella@scienze.uniroma2.it (G.P.); leonardo.severini@uniroma2.it (L.S.); 2 Department of Enterprise Engineering ‘Mario Lucertini’, University of Rome ‘Tor Vergata’ and INSTM RU Roma-Tor Vergata, Via del Politecnico 1, 00133 Rome, Italy; lorenzo.paleari@uniroma2.it (L.P.); bragaglia@ing.uniroma2.it (M.B.); fnanni@ing.uniroma2.it (F.N.)

**Keywords:** Opadry, ageing, PVA, titanium dioxide, aluminium hydroxide, photo-degradation, thermal degradation

## Abstract

Most tablets put on the market are coated with polymers soluble in water. The Opadry II 85 series from Colorcon Inc., is a family of PVA-based products marketed since the 1990s. Despite numerous publications on the properties of PVA, to date, limited work has been undertaken to determine the physico-chemical parameters (i.e., UV light, high temperature, and relative humidity) that could affect the performance of PVA-based coatings. To this end, we performed artificial ageing processes on samples made of Opadry Orange II or of some selected components of this coating and analysed them by means of a multidisciplinary approach, using, for example, FTIR, NMR, rheology, and DMTA measurements. In this way, we analysed the influence of the critical components of the Opadry Orange II formula, such as titanium dioxide and aluminium hydroxide, on the coating characteristics under ageing conditions.

## 1. Introduction

Tablets are the most commonly prescribed solid dosage formulations due to their precision of dosing, portability, manufacturability, enhanced stability, and consistent biopharmaceutical performance. Furthermore, tablets allow for greater patient compliance with therapy. Many tablets put on the market are coated with natural or (semi-)synthetic polymers because coating improves their aesthetic, handling, and product stability, as well as the resistance of tablets to damage such as chipping and erosion due to shipping and handling [1,2]. In addition, it can help the patient recognise the tablet because of its unique colour, mask an unpleasant odor and/or taste, and facilitate swallowing. Usually, the composition of a common aqueous-based coating system includes, besides the film-forming polymer, also plasticisers, pigments, glidants, and other ingredients such as surfactants, stabilisers, and anti-foaming agents. The aforementioned additives are added to increase the stability and processability of the coating system [3]. However, these additives can interact with the polymer and, over time, can lead to changes in the product performance [1].

Poly(vinyl alcohol) (PVA) is a synthetic poly-hydroxyl, nontoxic, biocompatible, and biodegradable polymer, commonly used as an aqueous-based film-forming polymer ingredient in different coating systems [3]. It is indeed capable of generating film coatings that are stable and soluble [1]. Moreover, it provides high tensile strength, abrasion resistance, and a smooth finish to tablets [1,2]. Furthermore, PVA-based films are known to have relatively low moisture vapor and oxygen permeability, providing sufficiently high oxygen barriers for most drug products [3]. It should be considered that the fact that PVA-based films have an inherently low water vapor transmission rate does not guarantee that tablets coated with PVA-based coatings will be stable in moisture-rich environments. As is known in the literature, the final stability of a solid dosage form is dependent on both the formulation and processing variables, including the core excipients and manufacturing conditions (e.g., relative humidity and temperature), film-coating formulation and process parameters, as well as also the packaging materials [3]. The Opadry series (Colorcon Inc. West Point, PA USA) is a family of PVA-based products that was commercialised in the mid- to late 1990s. Several studies were carried out on the two main PVA-based coating systems, Opadry AMB [4,5,6,7] and Opadry II [1,2]. The aqueous moisture barrier of Opadry AMB was thoroughly investigated, and even though the formulation was optimised to provide a low water vapor transmission rate, Bley et al. have demonstrated that the water uptake of Opadry AMB is related to structural polymer changes (glassy-to-rubbery state transition) after storage at room temperature and elevated relative humidity [4]. An improvement in Opadry AMB is the Opadry II 85 series; studies on the Opadry White II coating system, for example, proved that PVA formed an insoluble film under acidic conditions, and that the rate and extent of the formation of insoluble films depended on the acid concentration [1,2].

However, despite numerous publications on the properties of PVA, to date, limited work has been carried out to determine the physico-chemical parameters affecting the features of PVA-based coatings. The objectives of our studies were to evaluate the effect of critical parameters, such as UV light, high temperature, and relative humidity, on the final performance of the Opadry II coating system, which, in turn, can lead to the failure of the dissolution specifications. Importantly, as Opadry Orange II 85F is composed of PVA and polyethylene glycol (PEG), talc, titanium dioxide (TiO_2_), and Aluminum Lake Sunset Yellow #6, in this study, to define the role of the additives and its concerted action in the stability of this coating, we have performed the same study on samples containing only selected compounds. More in detail, we artificially aged (photochemically or thermally) and characterised Opadry Orange II 85F as a model PVA-based coating formulation in this study. Disintegration tests were performed on *ad hoc* prepared tablets, unaged or artificially aged, to correlate the chemical ageing and coating performances. The results were obtained using spectroscopic and non-spectroscopic techniques such as FTIR, NMR, and rheology.

## 2. Results and Discussion

To investigate the effects of ageing on the Opadry II performance, and, in turn, on its components, several samples were prepared and analysed. The composition of the samples was selected of the basis on their potential role in ageing [8]. To this end, the samples reported in Table 1, prepared as described in the Materials and Methods section were used; for their preparation, in all cases, 85% hydrolised poly(vinyl) alcohol (PVA) was used (see Section 3 and Figure S1). Images of them are in Figure S2. The samples were then subjected to thermal or UV ageing and the effects were evaluated.

### 2.1. Thermal Ageing

Several studies have initially been conducted on the behavior of the samples in thermal ageing. For this purpose, the samples were incubated for 40 h at T = 70 °C (see Section 3) unless otherwise stated.

**Figure 1:**
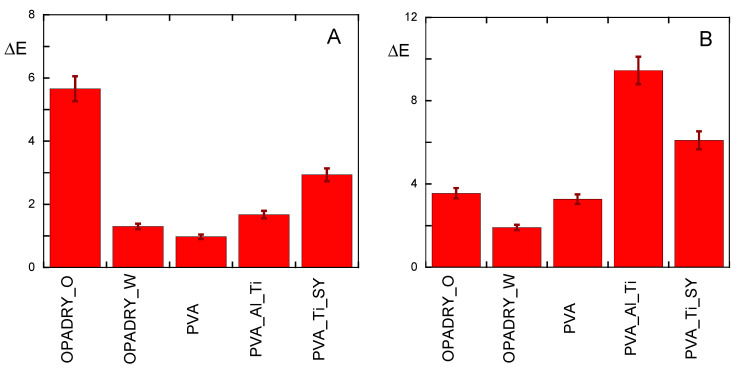
Colorimetric variations in (**A**) thermally or (**B**) UV-aged samples. Data are reported using non-aged samples as references.

First, the macroscopic variations have been evaluated in terms of the color [9] or hydrophobic properties of the sample surfaces [10,11]. Colorimetric variation was investigated because it could represent a problem from a commercial point of view as tablets coated with an aged coating could be less attractive to the consumer, but also, importantly, because of the presence of chemical reactivity. In Figure 1A, the colorimetric variation (ΔE) of the samples, due to thermal ageing, is reported; as shown, in all cases, ageing causes a change in the optical quality of the coating, optical changes that are more evident in the presence of the Sunset Yellow # 6 dye and/or titanium dioxide.

In Table 2, the contact angle measured on the surfaces before and after ageing has been reported, while, as an example, images of the contact angle measured on PVA_Al_Ti before and after ageing are shown in Figure S3.

**Figure 2:**
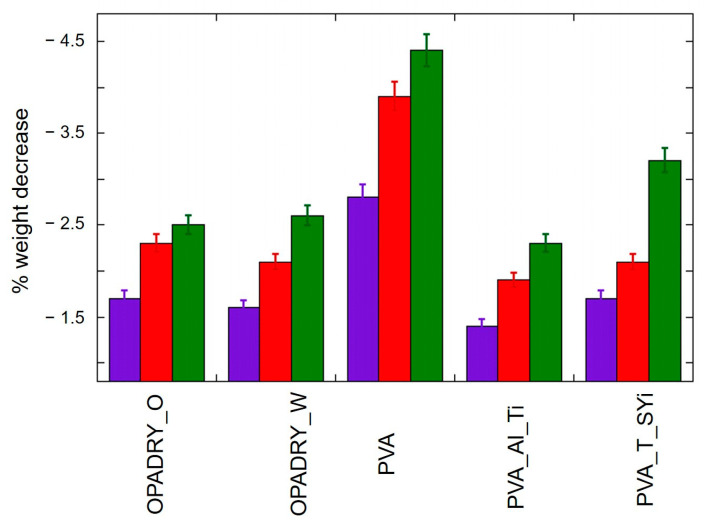
Sample weight decrease: not aged (violet), photo-aged (red), and thermally aged (green) after drying for 96 h at RH = 1%.

It is interesting to note that thermal (T) ageing causes a decrease in the measured contact angle, indicating an increase in the wettability of the samples. This result is confirmed by the weight decrease measurements reported in Figure 2. In fact, as shown in the Figure, the weight measurements indicate that the water uptake by the coating increases after thermal ageing; the weight decrease due to drying at RH = 1% for 96 h is enhanced for the aged materials, indicating that at room temperature, they are more prone to adsorb humidity. One possibility lies in the loss of acetate ester groups due to thermal ageing, which reduces the overall hydrophobicity of the PVA polymers and favors water uptake [12]. It should be noted that samples containing not only PVA but also other additives (e.g., TiO_2_) tend to adsorb less water than PVA, as already reported in the literature [12,13].

**Figure 3:**
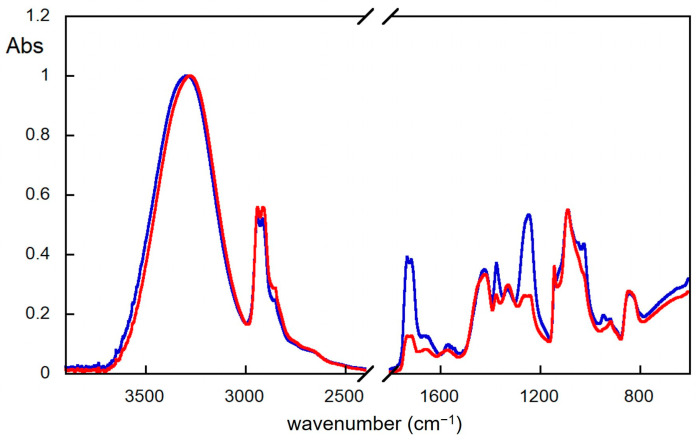
FTIR spectra of PVA film samples not aged (blue) and thermally aged for 72 h (red). Spectra are normalised on the most intense band for clarity.

In the case of thermal ageing, the hydrolysis of the ester groups [14] could also explain the results of FTIR measurements. Indeed, as shown in the FTIR spectra reported in Figure 3, in the case of the PVA thermally aged for 72 h, a decrease in the bands due to C=O stretching (at about 1730 cm^−1^) and due to C-O stretching in the esters (localised at 1240 cm^−1^) occurs. At the same time, an increase in the relative band at 1089 cm^−1^ (assigned to C-O stretching in the alcohol groups) is observed. These results indicate a decrease in the ester groups in the PVA backbone and a concomitant increase in the alcoholic ones.

**Figure 4:**
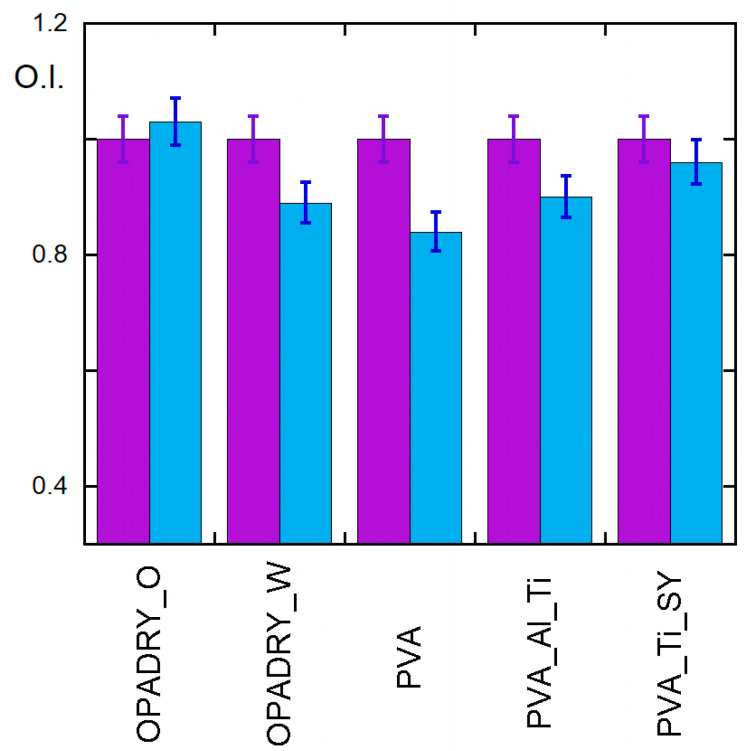
Oxidation index (O.I.) of samples under study before (violet) and after (light blue) thermal ageing. For clarity, data are normalised with respect to non-aged samples.

A first evaluation of reactivity of the samplesis derived from FTIR spectra, determining the ratio (called O.I) of the intensity of the band at 1730 cm^−1^ (I_CO_) to the one at 2926 cm^−1^ (I_CH_). The first is due to the presence of carbonyl and carboxyl groups (which are in esters, acids, aldehydes, and ketones), while the second is univocally attributable to CH_2_ asymmetric stretching [15,16], and therefore its intensity is almost independent from the reactions occurring in the samples. Results are shown in Figure 4.

**Figure 5:**
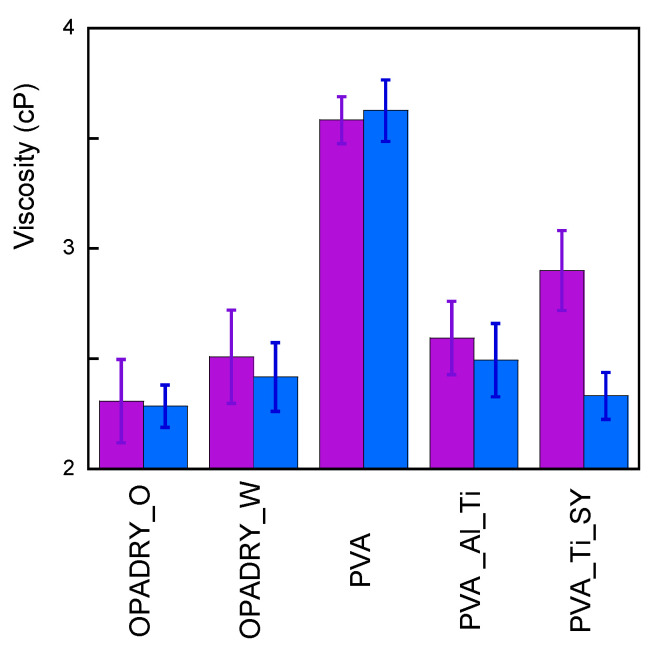
Viscosity of non-aged (violet) and T-aged (light blue) samples under study.

The data indicate in all samples a decrease in the O.I. that is almost independent of the composition (besides the presence of PVA) of the samples and that, therefore, can be attributed to the hydrolysis of the ester units in the PVA. This finding is confirmed by the NMR results performed on a thermally aged PVA sample (Figure S4), which indicate an increase in nonacetylated polymer of up to 87.8%. The increase in ageing time causes a further improvement in the hydroxylation of samples, as shown in Figure S5. Curiously, the O.I. variation in the 72 h T-aged samples is much less in the samples containing TiO_2_ and SY (that are Opadry Orange and PVA_Ti_SY ones). This finding may indicate that further processes can be taken into account and it deserves further investigation, which will be discussed in a future article. The hydrolysis of the ester units also explains the increased water uptake of the samples after ageing. In fact, according to the literature, deacetylated PVA is more hydroscopic than acetylated PVA [17]. The independence of the viscosity values in thermal ageing, as shown in Figure 5, confirms the FTIR results; thermal ageing does not determine severe reticulation or depolymerisation. However, it must be highlighted that the viscosity is influenced by the molecular weight, but the variation owing to polymer degradation might not be detectable.

### 2.2. Photochemical Ageing

As reported in the literature [18,19], photochemical (UV) treatments can lead to severe changes in the PVA and PVA-based systems, leading to PVA chain breakage and the formation of carbonyl products [18,19] as well as crosslinks [20,21]. The latter are due to the formation of highly reactive hydroxyl or oxy radicals, which can initiate the chemical reactions leading to the formation of covalent bonds (C-C and C-O) between chains. These changes lead to different macroscopic features with respect to thermal ageing, as evidenced by the contact angle results reported in Table 2. Indeed, in contrast to thermal ageing, the contact angles obtained do not increase considerably with respect to those of unaged ones, indicating that no great changes in wettability are induced by photochemical ageing. These findings are supported by the weight decrease results obtained by drying the non-aged or aged samples, shown in Figure 2; thesedata indicate indeed that even if the water uptake by the coating increases after ageing, it is lesser in the case of UV ageing with respect to T aging.

**Figure 6:**
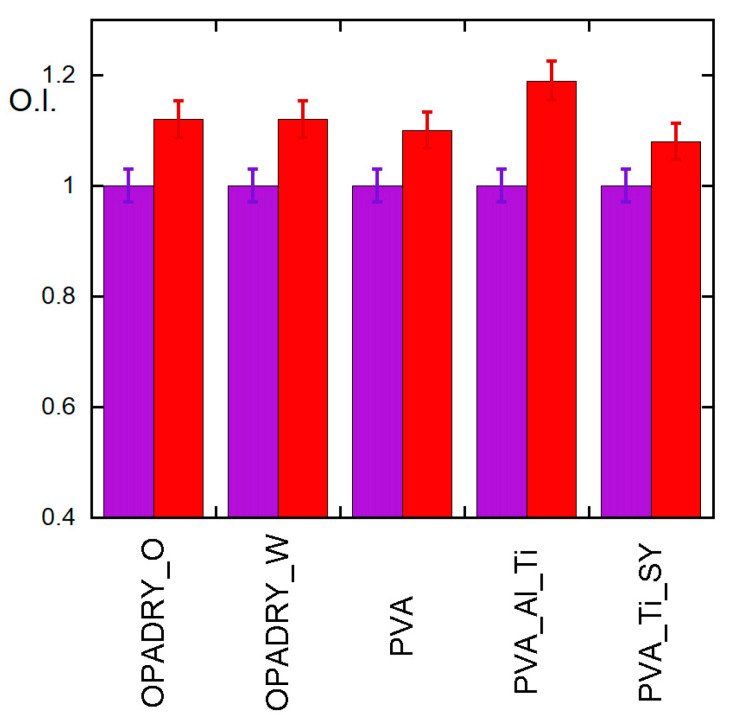
Oxidation index (O.I.) of samples under study before (violet) and after (red) UV ageing. For clarity, data are normalised with respect to non-aged samples.

Furthermore, as shown in Figure 1B, first of all, UV ageing causes a change in the optical quality of the coating that is very important in the PVA_Ti_Al sample, presumably due to the synergic effect of TiO_2_ and Al(OH)_3_ on the ageing of the PVA. TiO_2_ [18,22,23,24,25] is indeed known to induce photodegradation, while aluminium ions can coordinate the hydroxyl groups of the PVA, thus facilitating oxidation and crosslinking between chains [21,26]. This hypothesis may also explain the slight higher increase in the O.I. data for the PVA_Ti_Al sample (Figure 6). At the same time, the presence of the Sunset Yellow #6 dye, which is capable of absorbing UV light (as shown in Figure S6) in the PVA-Ti-SY samples seems to protect against UV ageing.

**Figure 7:**
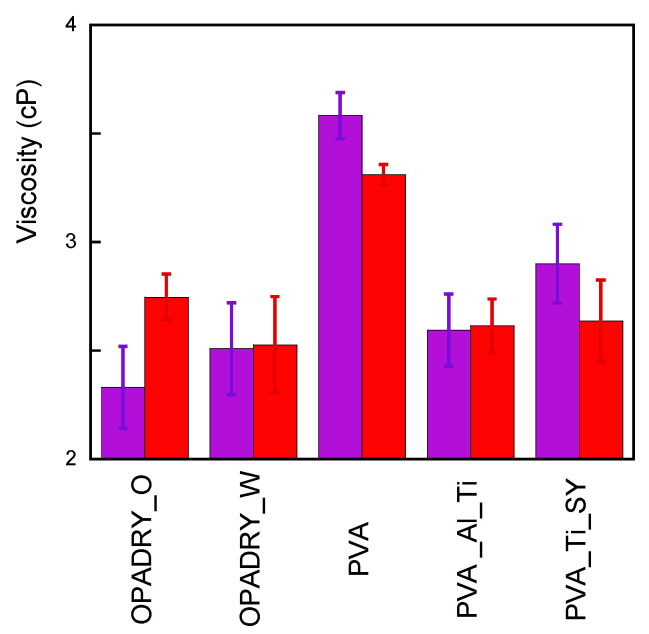
Viscosity of non-aged (violet) and UV-aged (red) samples under study.

Viscosity measurements have also been performed on the samples under investigation (Figure 7). It is interesting to note that, while the viscosity decreases in the PVA sample, it remains substantially constant or slightly increases in almost all samples; this suggests that while in the previous samples the depolymerisation process prevails, in the others, a concurrence of polymer chain scissions and cross-linking could occur.

**Figure 8:**
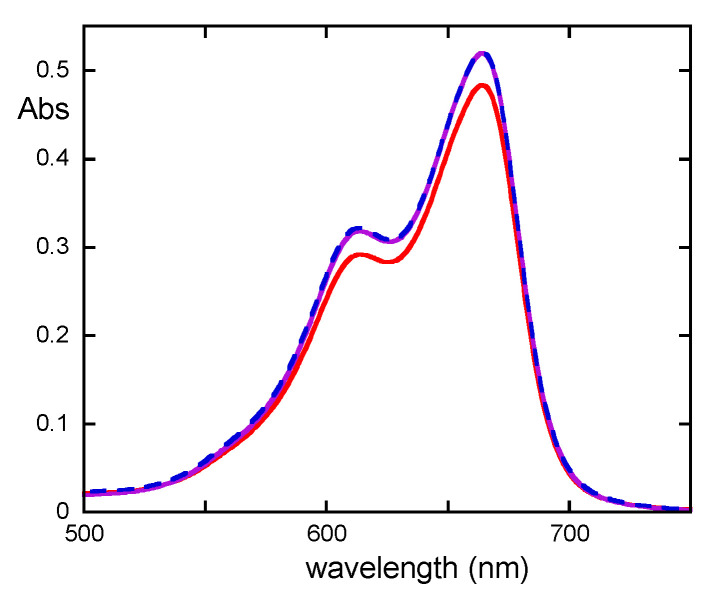
UV-Vis spectra of MB solution in the absence and in the presence of non-aged (violet), T-aged (blue), and UV-aged (red) PVA films.

Finally, in the case of PVA films, the effect of ageing has also been tested using a methylene blue (MB) test. MB is a blue dye that is capable of forming a colorless complex with acidic molecules [27]. Therefore, it is possible to follow the decrease in the band centered at 664 nm (attributable to the dye) to determine the presence of acid molecules in the sample. As shown in Figure 8, the decrease in the MB band occurs only with the UV-aged PVA film. The reported results strongly suggest that, in the case of UV ageing, due to chain scission, high-molecular-weight non-volatile acid molecules are formed, as reported in the literature [19]. In the case of thermal ageing, instead, because of the hydrolysis of the ester groups, acetic acid, which is volatile and thus is not present in the MB solution, is produced.

### 2.3. Further Studies on Opadry_O-Based Samples

As explained in the Introduction section, Opadry_O is widely used in the pharmaceutical industry. Furthermore, it contains all the compounds (as TiO_2_ and aluminium hydroxide) that play a role in ageing. To this end, samples based on this colored coating were further investigated.

**Figure 9:**
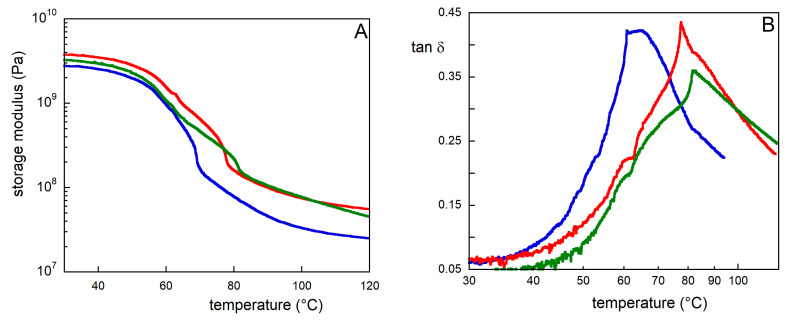
(**A**) Storage modulus and (**B**) dumping factor for non-aged (blue), thermal aged (green) and photo-aged (red) Opadry_O samples.

First, dynamic mechanical thermal analyses (DMTA) were performed on the Opadry_O film to determine the mechanical performance of the coating. In Figure 9, reported is a comparison of the storage modulus as a function of temperature for neat and T- or UV-aged samples. Both kinds of ageing produce a slight increase in the stiffness of the Opadry_O film (to a lesser extent in the T-aged ones), as the modulus varies from 2.9 GPa to 3.1 and 3.9 GPa for T- and UV-aged samples, respectively. In fact, during thermal treatment, the sample is subjected to thermo-oxidative degradation and hydrolysis, accelerating the conformational changes in the macromolecules, with a consequent increase in stiffness [28]. Also, during photoageing, a photooxidative degradation mechanism takes place and the formed radicals react, causing a higher amount of cross-linkingand determining a higher stiffness value compared to the thermally aged sample. Furthermore, the maximum value of the dumping factor, tan δ, related to the glass transition temperature also changes to a higher temperature after ageing, thus indicating the occurrence of crosslinks among the PVA chains due to ageing. This behavior corroborates the results obtained from the FTIR analysis and can be then ascribed to the crosslinking phenomenon which takes place during thermal and photo-irradiation, as already explained [29].

In principle, a coating formulated for immediate release should not have a measurable effect on the pharmaceutical properties of coated tablets and thus on their dissolution profiles. However, it has been proved that the coating can control the dissolution rate. As reported in the literature [30], several stages are involved in the dissolution of the coating layer, which activates the tablet disintegration process, such as hydration, swelling, and then the entrance of water into the core tablet. It has been reported that the wetted coating can act as a barrier to the diffusion of water in the core, influencing the disintegration and therefore the dissolution rate [30]. To determine the effect of ageing on the performance of Opadry_O coating, tablets made of microcrystalline cellulose, crospovidone and sodium stearyl fumarate were prepared and coated with a Opadry_O coating; the disintegration times of these tablets were measured (Table 3). In the case of photo-ageing, degradation may lead to cross-linking, and although cross-linked PVA films may be insoluble [31], the data collected from disintegration tests on the coated tablets showed that the extension of this reaction was not sufficient to impair the performance of Opadry_O in the disintegration medium. The disintegration tests indicate indeed that the start and ending disintegration times are comparable to those of the unaged sample. This is not the case for thermal ageing, in which the stiffening and wettability increases lead to a major slowing down of the disintegration times.

## 3. Materials and Methods

### 3.1. Materials

The coating formulations of Opadry Orange II 85F and Opadry White II 85F as well as Aluminum Lake Sunset Yellow #6 were obtained from Colorcon, Inc. (West Point, PA, USA). The partially hydrolysed poly(vinyl alcohol) (PVA, average Mw 30,000, 85% hydrolyzed), Al(OH)_3_, and TiO_2_, as well as all the other reagents, were purchased from Merck (MercK KGaA, Darmstadt, Germany). In all cases, bidistilled water (Millipore, Billerica, MA, USA) was used in the preparation of solutions. The degree of deacetylation has been obtained using NMR experiments [32] (see SI and Figure S1) and has been ascertained to be 85.5%.

### 3.2. Preparation of the Aqueous Casting Films

The Opadry II White 85F or Opadry II Orange 85F (1.5 *w*/*w*) suspensions were prepared in deionised water (Millipore, Merck KGaA, Darmstadt, Germany). The suspensions were stirred for 1 h before casting into polystyrene dishes and subsequently dried at room temperature for 48 h. The solution of PVA (15% *w*/*w*) and suspensions of PVA-Al(OH)_3_-TiO_2_ and PVA-TiO_2_-Aluminum Lake Sunset Yellow #6 were also prepared, casted in polystyrene dishes, and dried at room temperature. The list of samples, their composition, and their abbreviation are reported in Table 1. The percentages of the components were selected based on the coating formulation examples disclosed in BPSI Holdings, Inc. patent US 6,448,323 [33].

### 3.3. Preparation of the Coated Tablets

The tablet cores (819 ± 4 mg) were manufactured using direct compression using a manual tablet press equipped with stamps (Unico S.p,A, Milan, Italy). The following excipients were used: microcrystalline cellulose, crospovidone, and sodium stearyl fumarate in an 85:13:1. ratio (*w*/*w*). An aqueous coating dispersion was prepared according to Colorcon’s recommended procedures. The Opadry Orange II was prepared by mixing the powders (20% *w*/*w*) in distilled water at room temperature. The tablets were covered via immersion in the Opadry Orange suspension under stirring and dried at room temperature for two days. The coating thickness was measured using a caliper to be 0.10 ± 0.02 mm.

### 3.4. Ageing of the Samples

Photochemical ageing was obtained by exposing the samples for three hours at room temperature to light in a photoreactor (Photochemical Multirays, Helios Quartz S.R.L, Cambiago, Italy) equipped with 10 UV-Vis lamps whose emission was centered at λ_em_ = 365 ± 50 nm. Thermal ageing was performed in a customised climate chamber by exposing the samples to T = 70 °C and RH = 70% for 40 h (unless otherwise stated). The thermal ageing temperature of 70 °C was chosen because it is close to the transition temperature of Opadry Orange II (about 65 °C) and close to the coating temperature suggested in the information on Colorcon’s Opadry product.

### 3.5. Water-Desorbing Experiments

The non-aged, thermally aged, and photo-aged samples were cut into small rectangular pieces (about 20 × 20 × 0.2 mm^3^) and weighed (about 85 mg). The samples were hydrated at a RH of 80% for 48 h, weighed, and then dried at a RH of 1% until a constant weight was obtained (96 h). The weight loss (*W_d_*), expressed as a percentage, was calculated using the following equation.

$$W_{d}=\left[\frac{W_{2}-W_{1}}{W_{1}}\right]\times 100$$

where *W*_1_ is the sample weight before drying and *W*_2_ is the sample’s weight after drying.

### 3.6. Rheological Measurements

Viscosity tests were performed on a 4% (*w*/*v*) solution of each sample. Rheological tests were carried out using a cone plate rheometer (Malvern Kinexus Lab+, Spectris, London, UK) at room temperature in a frequency sweep configuration (1–100 s^−1^).

### 3.7. UV-Vis and FTIR Absorption Spectroscopy

The solutions of PVA in the presence of methylene blue (MB) at pH = 8.0 (Tris buffer, 0.1 M) were characterised before and after the different treatments using UV-vis absorption spectroscopy using a Cary 100 scanner (Agilent, Billerica, CA, USA). All the absorption experiments were performed using quartz cells with a 1 cm optical path length (Hellma Italia srl, Milan, Italy).

The FTIR spectra of the samples were obtained using a Thermo Fisher spectrometer mod. iS50 (Thermo Fischer Scientific, Madison, WI, USA), equipped with a single-reflection ATR diamond cell. For each spectrum, 32 scans with a resolution of 4 cm^−1^ were collected. Unless otherwise stated, all the samples were conditioned for 1 week under the same conditions of temperature (20 ± 0.5 °C) and relative humidity (45 ± 2%), as established by the ISO 1 standard [34], and then analysed.

### 3.8. NMR Spectroscopy

The NMR experiments were performed in deuterated water at 25 and 70 °C and recorded using a Bruker ADVANCE spectrometer operating at 700 MHz for ^1^H, equipped with a 5 mm inverse TXI probe and z-axis gradients. The ^1^H-NMR experiments were acquired with a spectral window of 15.9 ppm (carrier frequency at 4.7 ppm) using a relaxation delay of 8 s, an acquisition time of 0.7 s, and a 30° pulse to ensure full longitudinal magnetisation recovery between scans. All the data were processed using TopSpin v. 3.6.4 using a sine-squared window function and zero filling.

### 3.9. Colorimetry Experiments

The colorimetry results were obtained using an EOPTIS Digital Handheld Colorimeter (mod. CLM-194, EOPTIS, Trent, Italy), using a D65 illuminant and a 10° observer. For each sample, at least three measurements were performed.

### 3.10. Dynamic Mechanical Thermal Analysis

The viscoelastic behaviour of the samples was studied using dynamic mechanical thermal analysis (DMTA, Triton, Tritec, 2000, Arakawa City, Japan). The tests were carried out on film samples with a length of 20 mm and width of 5 mm, in the temperature range of 25–120 °C, in tensile configuration, at a frequency of 1 Hz, and imposing a displacement of 0.05 mm.

### 3.11. Disintegration Tests

The disintegration tests were performed on a laboratory-customised apparatus following the specifications of the European Pharmacopoeia [35]. Briefly, 6 coated tablets were placed in six open-ended tubes closed with a mesh (2 mm in size) on the bottom. The tubes were then kept in periodic motion in a 1 L beaker filled with HCl at pH 2.0 for 10 min. During the test, the disintegration time of the coating of tablets was registered.

## 4. Conclusions

In this work are presented experiments that aimed to characterise the stability of PVA-based coatings like Opadry White II 85F and Opadry Orange II 85F. The data obtained indicate that the sample consisting of PVA only underwent degradation after photo- and thermal ageing, and this phenomenon was exacerbated by the presence of TiO_2_ and Al(OH)_3_ in the coating formulation. More specifically, thermal ageing leads principally to a hydrolysis of the PVA constituting the Opadry coating and a change in the wettability of the coating itself, while UV ageing leads to depolymerisation and/or reticulation reactions, leading to a greater stiffness, high viscosity, and higher content of oxidised products than non-aged coatings. Moreover, the desorption humidity assays performed on the casting coating showed high moisture sensitivity for the thermal and photo-aged coatings. Finally, the data collected suggest that the coating should occurr through a strict control over both temperature and relative humidity, which have been shown to greatly influence the final performance of the coated tablets.

## Figures and Tables

**Table 1:** Samples under study.

Name	Composition	Abbreviation
Opadry White II	PVA, PEG 3000, lecithin (soy), talc, TiO_2_	Opadry_W
Opadry Orange II	PVA, PEG 3000, talc, TiO_2_, Sunset Yellow #6	Opadry_O
PVA	Poly(vinyl alcohol)	PVA
PVA-Al(OH)_3_-TiO_2_	PVA/Al(OH)_3_/TiO_2_ = 76/4/20 (*w*/*w*)	PVA_Al_Ti
PVA-TiO_2_-Sunset Yellow #6	PVA/ Sunset Yellow #6/TiO_2_ = 76/4/20 (*w*/*w*)	PVA_Ti_SY

**Table 2:** Contact angles measured on non-aged, thermally aged (T-aged), and photochemically aged (UV-aged) samples.

Sample	Non-Aged	T-Aged	UV-Aged
Opadry_O	(42 ± 1)°	(38 ± 2)°	(43 ± 1)°
Opadry_W	(44 ± 1)°	(35 ± 1)°	(49 ± 2)°
PVA	(55 ± 2)°	(47 ± 1)°	(57 ± 2)°
PVA_Al_Ti	(61 ± 1)°	(37 ± 1)°	(61 ± 2)°
PVA_Ti_SY	(46 ± 2)°	(42 ± 2)°	(53 ± 1)°

**Table 3:** Average start and ending time of tablet disintegration (n tablets = 6).

Disintegration Time (s)	Not Aged Tablet	T Aged Tablet	UV Aged Tablet
start-end	6–34	35–70	4–35

## Data Availability

Data are contained within the article and supplementary materials.

## Supplementary Materials

The following supporting information can be downloaded at https://www.mdpi.com/article/10.3390/molecules29020386/s1. Additional spectroscopic data concerning the aged samples are available. Figure S1: ^1^H-NMR spectrum of PVA; Figure S2: Images of the film samples under study; Figure S3: Drop of water on (A) unaged and (B) thermally aged PVA_Al_Ti samples; Figure S4: ^1^H-NMR spectra of unaged PVA and thermally aged PVA; Figure S5: Oxidation index (O.I.) of samples under study before and after 40 or 72 h of thermal ageing; Figure S6: UV-Vis absorbance spectrum of the Sunset Yellow # 6 dye at pHs = 2.0, 5.5, and 7.5.
