# Supplementary material for: Investigation into the Influence of the Process Parameters on the Stability of a Poly(Vinyl)-Alcohol-Based Coating System
Source: Molecules. 2024 Jan 12;29(2):386. doi: 10.3390/molecules29020386 (PMC10820689; doi:10.3390/molecules29020386)
Supplement: Supplementary file 1 [file molecules-29-00386-s001.zip › molecules-2718134-supplementary.pdf]

Materials and Methods

NMR experiments

Polyvinyl alcohol (PVA) powder was dissolved in 0.60 mL of D_2O. The sample was analyzed by Nuclear Magnetic Resonance (NMR) spectroscopy. The acquired proton spectrum (Figure S1) shows three regions that can be assigned to CH (4.0-3.0 ppm), CH_3-CO (2.1-1.9 ppm), and CH_2 (1.9-1.4 ppm) groups.

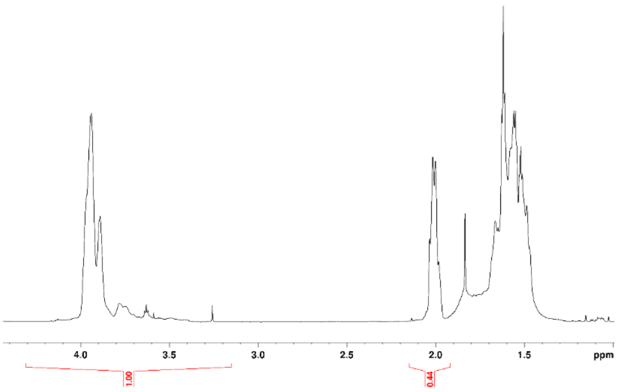

Figure S1. ^{1}H-NMR spectrum of PVA.

The percentage of non-acetylated PVA can be calculated by the following equation:

$$PVA\% = 100\left(1 - \frac{Int_{CH3}/Int_{CH}}{3}\right)$$

Where Int_{CH3} and Int_{CH} refer to the integral values of the CH_3-CO and CH regions, respectively.

The result for the sample analyzed was equal to 85.5%.

Moreover, to understand the temperature stability of the sample, it was subjected to a 60-hours of artificial degradation by increasing the temperature to 70°C, but no differences were observed on the ^{1}H-NMR spectrum.

Results and Discussion

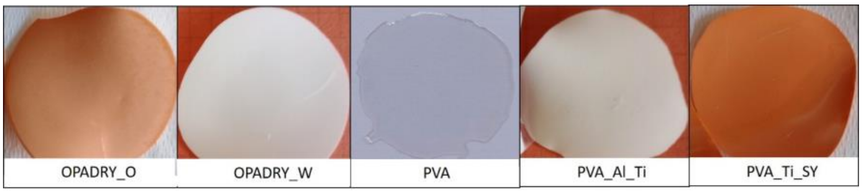

Figure S2. Images of the film samples under study.

Thermal ageing

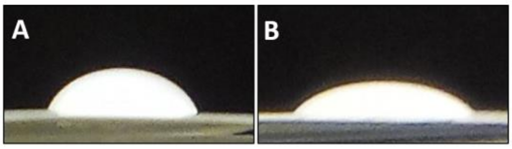

Figure S3. Drop of water on (A): unaged; (B): thermally aged PVA_Al_Ti sample.

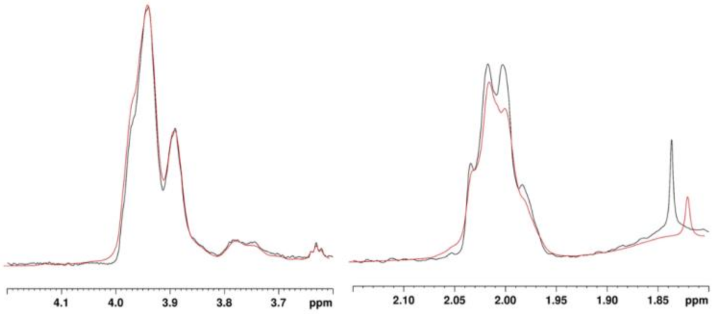

Figure S4. ^{1}H-NMR spectra of not aged PVA (black) and thermally aged PVA (red). A decrease in area of the CH3-CO-related signals of the artificially aged PVA can be seen once the CH signals (4.0-3.0 ppm) of the two polymers are placed at the same height.

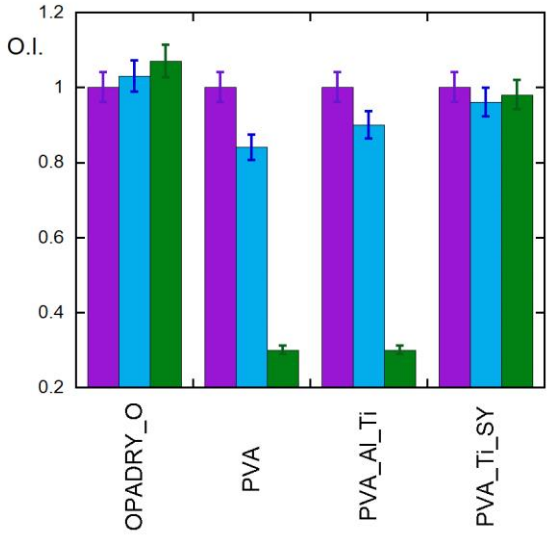

Figure S5. Oxidation Index (OI) of samples under study before (violet) and after 40 hs (light blue) or 72hs (green) of thermal ageing. For clarity, data are normalized with respect to not aged samples.

Photochemical ageing

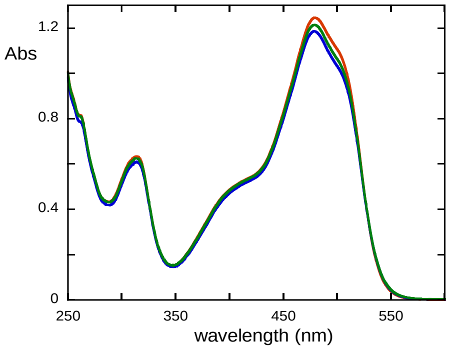

Figure S6. UV-Vis absorbance spectrum of the Sunset Yellow # 6 dye at pH = 2.0 (red), 5.5 (blue) and 7.5 (green).
